# Prevalence of dyslipidemia among teachers in China: a systematic review and meta-analysis

**DOI:** 10.3389/fpubh.2024.1425387

**Published:** 2024-08-27

**Authors:** Xiaoxue Wei, Feng Ouyang, Yang Liu, Qingfeng Du

**Affiliations:** ^1^Faculty of Medicine, Macau University of Science and Technology, Macau, China; ^2^The Seventh Affiliated Hospital of Southern Medical University, Foshan, China; ^3^School of Traditional Chinese Medicine, Southern Medical University, Guangzhou, China

**Keywords:** Chinese teachers, dyslipidemia, prevalence, meta-analysis, systematic review

## Abstract

**Objective:**

To systematically analyze the current situation with dyslipidemia among teachers in China, to provide guidance for lipid management and prevention of ASCVD.

**Design:**

A systematic review and meta-analysis of the prevalence of dyslipidemia among teachers in China.

**Methods:**

We searched via 9 databases for studies published between June 1, 1996, and July, 25, 2024. The article were evaluated by the Joanna Briggs Institute (JBI) Article Quality Assessment Tool (2016) in Australia. RevMan5.0 and R4.3.1 software were used for statistical analysis to calculate the OR and RR values and the 95% confidence intervals. This systematic review and meta-analysis were reported in line with the PRISMA guidelines.

**Results:**

A total of 12 cross-sectional studies from 10 provinces (regions) were included, with the sample of 24,851, including 12,626 males and 12,198 females, the average age of about 40 (1,036 were aged ≤30, 5,872 were aged 30–40, 3,854 were aged 40–50, 4,607 were aged 50–60, and 3,425 were aged ≥60), including 9,114 people with dyslipidemia. The overall prevalence of dyslipidemia among teachers in China was 38% (*p* < 0.01, 95% CI (1.27–1.95)). The prevalence of hypertriglyceridemia was 21.6% (*p* < 0.01, 95% CI (1.05–1.50)), that of hypercholesterolemia was 13.3% (*p* < 0.05, 95% CI (0.98–1.34)), that of hyper-LDL-Cemia was 9.4% (*p* < 0.01, 95% CI (1.04–1.59)), and that of hypo-HDL-Cemia was 4.3% (*p* = 0.25, 95% CI (0.61–6.52)). The heterogeneity of dyslipidemia among teachers of the different sexes was I^2^ = 92% (*p* < 0.01). The overall prevalence of dyslipidemia, as well as that of high TC, high TG, and high LDL-C levels, was greater in female teachers than in male teachers (df = 10, 95% CI:1.35–1.52, *p* < 0.01). The heterogeneity of dyslipidemia among teachers of different ages was I^2^ = 74% (*p* < 0.01), and the risk was lower for aged <50 years than those aged ≥50 years (df = 7, 95% CI: 0.38–0.44, *p* = 0.04). The year, region, school type, and these factors showed no effect on the prevalence of dyslipidemia (*p* = 0.7353).

**Conclusion:**

The prevalence of dyslipidemia in the teacher population in China is high and tends to increase with age. We should pay attention to the health management of the teachers, which can be done by appropriately adjusting the educational model settings, increasing the programs on physical activities, promoting the improvement of healthy lifestyles.

**Systematic review registration:**

https://www.crd.york.ac.uk/prospero/, identifier [CRD42024567785].

## 1 Introduction

Cardiovascular disease (CVD) is one of the most important chronic noncommunicable disease (NCD) that seriously threatens human life and health globally and has become a major global public health problem (1, 2). CVD-related deaths account for 43.7% of total deaths worldwide every year. In 2022, the number of deaths caused by CVD in China reached 53%. CVDs account for 43.7% of all deaths globally every year (2). The number of deaths caused by CVDs in China reached 53.2% in 2022 (2). Dyslipidemia is closely related to atherosclerotic cardiovascular disease (ASCVD), being an initiating factor of ASCVD, and can increase the risk of morbidity and mortality of coronary heart disease and stroke by 3–5 times. However, China’s society as a whole still pays little attention to the prevention and control of lipid-associated diseases, and the prevalence of dyslipidemia continues to increase (3). In 2012, the total cholesterol, LDL cholesterol and triglyceride levels in China’s population (*n* = 60,0000) significantly increased compared with those in 2002, and the HDL cholesterol level significantly decreased (2). In recent years, the incidence of hyperlipidemia has risen significantly in all age groups (4, 5), and dyslipidemia has become an important public health problem in China (6). The State attaches importance to the treatment and management of hyperlipidemia, stressing that hyperlipidemia should be actively prevented and treated as an independent risk factor for cardiovascular and cerebrovascular diseases and incorporating the prevention and control of hyperlipidemia into the National Basic Public Health Program (7–10).

Dyslipidemia is a metabolism-related disease, the occurrence of which is strongly correlated with a poor lifestyle. High intake of sugar, fat, and a high-energy diet, drinking, obesity, staying up late, being sedentary, etc., are all risk factors. Teachers, as representatives of a large group of highly educated people in modern times, have a work nature that emphasizes brain power over physical strength, with greater mental stress and less physical activity, which lays hidden dangers to their health. Some studies have reported that teachers have a high incidence of dyslipidemic diseases (11, 12), domestic scholars have conducted research at different levels on the health of teachers in different regions of the country. The results show that metabolic-related diseases are the most important diseases affecting the health status of the teacher group and that diseases such as overweight, obesity, hyperlipidemia, fatty liver, hypertension, and hyperglycemia are highly prevalent in the teacher group; in particular, the detection rate of dyslipidemia is high. These chronic diseases are also high-risk factors for the development of cardiovascular and cerebrovascular diseases. Therefore, understanding the prevalence of dyslipidemia among brain workers, as the main group of dyslipidemia, and teachers, as the typical representatives of this group, is conducive to promoting the regular study of dyslipidemia among people, and taking effective management measures to control the level of blood lipids, which is of great significance for reducing the incidence of cardiovascular and cerebrovascular events and promoting the management of chronic diseases.

## 2 Materials and methods

### 2.1 Protocol and registration

We prospectively registered the protocol in the PROSPERO.

Database, and is awaiting approval. This systematic review and meta-analysis were reported in line with the Preferred Reporting Items for Systematic reviews and Meta-Analyses (PRISMA) statement guidelines (13) and are shown in Supplementary material 1.

### 2.2 Study design

PICO method was adopted to define our study question: P (Participants): Chinese teachers; I (Intervention): None; C (Comparisons): Non-teacher population; O (Outcomes): Blood lipid.

### 2.3 Inclusion and exclusion criteria

The inclusion criteria were as follows: (1) cross-sectional study; (2) the participants of the study were in-service teachers from different types of institutions; and (3) the main outcome indicators were the prevalence of dyslipidemia, including the overall prevalence and prevalence of different types of dyslipidemia. The exclusion criteria were as follows: (1) article with non-Chinese in-service teachers as subjects; (2) article without clear diagnostic criteria or a basis for dyslipidemia; (3) article with incorrect or incomplete data; (4) repeatedly published or updated article; (5) reviews or conference proceedings; and (6) article not in Chinese or English.

### 2.4 Search strategy

We searched the China Knowledge Network Infrastructure (CNKI), Wipro China Science and Technology Journal Database, Wanfang Full-text Database, China Medical Journal Full-text Database, Web of Science, PubMed, Springer Electronic Journals, BMJ Journals Collection, and CINAHL Ultimate Nursing full-text database for studies published between June 1, 1996, and July,25,2024 to define eligible studies. The search strategy uses topics words combine with free words. The following key words are searched: “hyperlipidemia, dyslipidemia, dyslipoproteinemia, blood lipids, blood fat, serum lipids, epidemiology, prevalence, incidence, detection rate, China, Chinese, teachers, teach staff.” Complete search strategies are shown in Supplementary material 2.

### 2.5 Article screening and data extraction

Two authors (WXX and OYF) independently evaluated the quality of these articles. When the evaluation opinions were inconsistent, the 2 researchers discussed their opinions based on the inclusion and exclusion until they reached unanimity; if they were unable to unify their opinions, they discussed and analyzed the article with the 3rd researcher and then selected it. When screening the articles, the first choice was to eliminate duplicates, then read the title and abstract, eliminate irrelevant articles, read the full text and then screen according to the criteria of screening. The extracted articles mainly included authors, year of article publication, sample size, sampling method, different types of schools, sex distribution, age distribution, regional distribution, diagnostic criteria for dyslipidemia, and major health outcome indicators.

### 2.6 Article quality assessment

The JBI Article Quality Assessment Tool (LQAT, 2016) was used to assess the initial inclusion of studies. The JBI LQAT for prevalence studies consists of nine entries (14) that assess the overall quality of prevalence studies in terms of the sampling method, the participants, data collection, analysis methods, etc., and each entry was graded as “yes,” “no,” “unclear,” or “not applicable.” Each entry was scored as “yes” (1 point), “no,” “unclear” or “not applicable” (0 points), and each entry was added to the total score (0 points). The scores were added together to obtain a total score (0–9). A score of 0–5 was considered low quality, 6–7 was considered medium quality, 8–9 was considered high quality, and finally, medium- to high-quality article was included.

### 2.7 Data synthesis and analysis

Data analyses used the meta package of R, version 4.3.1 and the meta package of. vRevMan5.0 to calculate the prevalence of dyslipidemia and its 95% CI. The heterogeneity among the included studies was analyzed by χ2 test (α = 0.100), and the heterogeneity was determined by I^2^. We used *p*-value and I^2^ to evaluate the heterogeneity. If *p* ≥ 0.100 and I^2^ ≤ 50%, the heterogeneity among studies was small, and the fixed effect model was adopted. If *p* < 0.100 and I^2^ > 50%, it shows that there is heterogeneity among studies, and the random effect model is adopted. Four subgroup analyses were conducted to explore factors impacting the result of the prevalence of dyslipidemia: sex, age, region (according to the commonly used regional geographical divisions in China), and school tier. Funnel plot and Egger’s test were used to evaluate publication bias. Sensitivity analysis was used to evaluate the stability and reliability of the analyses.

## 3 Results

### 3.1 Article screening results

**Figure 1:**
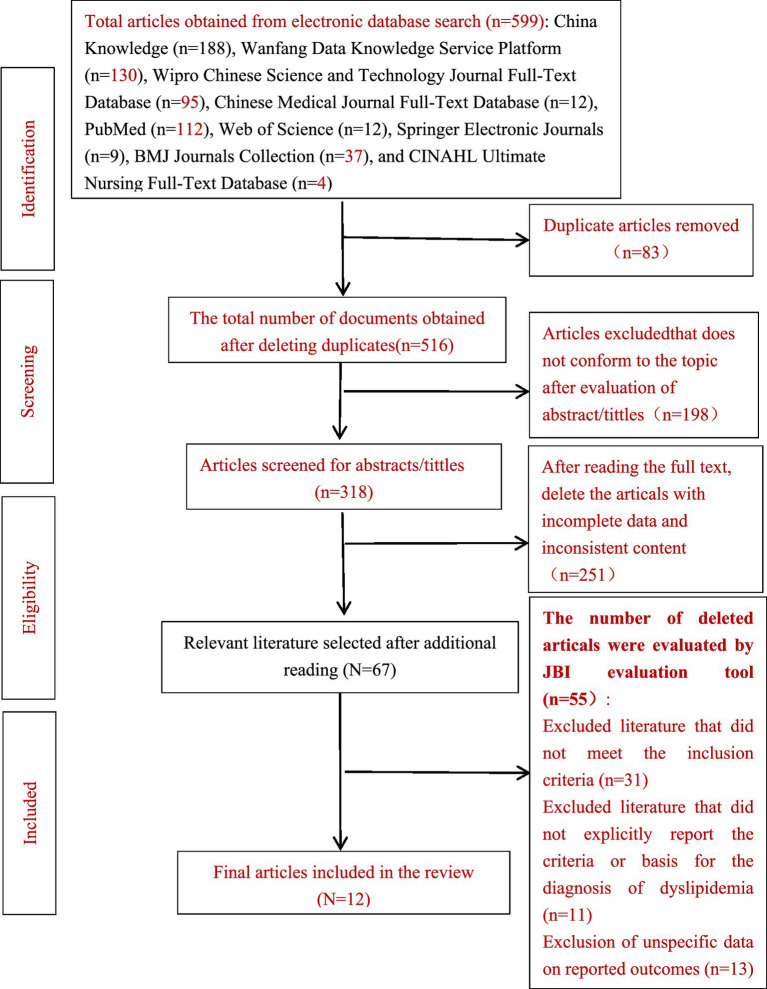
PRISMA flow diagram of study selection.

A total of 425 Chinese and 174 English articles were found, and 12 (15–26) cross-sectional studies on the prevalence of dyslipidemia among teachers were ultimately included and excluding conference papers and dissertations, as well as low-quality article (JBI article evaluation score of 5 or less). The 12 cross-sectional studies from 10 provinces (regions), with a total sample size of 24,851, including 12,626 males and 12,198 females, with an average age of about 40 (1,036 were aged ≤30, 5,872 were aged 30–40, 3,854 were aged 40–50, 4,607 were aged 50–60, and 3,425 were aged ≥60), including 9,114 people with dyslipidemia. The prevalence of dyslipidemia ranged from 18.73 to 60.3%. The flowchart of the article screening process is detailed in Figure 1.

### 3.2 Article quality evaluation

The JBI Article Quality Evaluation Tool was used to evaluate the quality of the initially included studies. There are 9 items in the evaluation index, with 1 point for each item and 9 points total. A score of 0–5 indicated low quality, 6–7 indicated medium quality, and 8–9 indicated high quality; 12 medium- and high-quality articles were ultimately included, as shown in Tables 1, 2.

**Table 1:** Basic characteristics of the Chinese teachers included in the analysis of the prevalence of dyslipidemia in China.

Author	Year of publication	Region	Sampling method	Sample size	Age/mean age	Sex (male/female)	Diagnostic criteria for dyslipidemia	Main outcome indicators: prevalence (%)
Xiezhen Yao (15)	2005	Dezhou City	Random sampling	958	26–78/42∙68 ± 14∙06	537/421	Dyslipidemia Prevention and Control Countermeasures Theme Group	① 37.06
Lihua Ma (16)	2012	Baoji City	Nonrandom sampling	1,650	20-70/unreported	954/696	Guidelines for prevention and treatment of dyslipidemia in Chinese adults	① 18.73
Shuxian Dai (17)	2008	Tibet	Nonrandom sampling	386	20-60/unreported	214/172	Internal medicine [M]. Beijing: People’s Health Publishing House,2004	① 22.02
Jian Hu (18)	2005	Guiyang City	Nonrandom sampling	1,402	27–83/56.75 ± 9.74	502/900	Recommendations for prevention and treatment of dyslipidemia (1997)	③ 39.2 ⑤ 3.3
Zhiqin Jiao (19)	2015	Harbin City	Chester sampling	1,249	25-60/unreported	627/622	Guidelines for the prevention and treatment of dyslipidemia in Chinese adults (2007)	② 60.3 ③ 16.6 ⑤ 7
Hong Li (20)	2017	Huaian City	Nonrandom sampling	1712	21-70/unreported	986/726		① 37.85
Guangbing Zhang (21)	2008	Mianzhu City	Nonrandom sampling	5,211	22–91/45.5 ± 9.4	2247/2964		① 25.9
Huajun Li (22)	2009	Linxiang City	Nonrandom sampling	1,236	23-72/unreported	712/524	Guidelines for the prevention and treatment of dyslipidemia in Chinese adults (2007)	② 12.08 ③ 17.70 ④ 10.11 ⑤ 8.71
YunWang (23)	2011	Harbin City	Nonrandom sampling	2,605	22-85/unreported	1483/1122		② 14.01 ③ 12.2 ⑤ 7.14
HongJing (24)	2010	Guyuan City	Nonrandom sampling	420	20–59/42.9	223/197		① 38.81
Xiaojuan Cha (25)	2008	Wuhu City	Random sampling	2026	30–85/56.5 ± 14.3	758/1268	WHO 1999 working definition	② 26.89 ③ 30.07 ⑤ 0.3.64
Liang Ran (26)	2011	Chongqing	Multilevel sampling	2,378	2,382/unreported	1357/1021	Internal medicine [M]. Beijing: People’s Health Publishing House,2004	① 47.05

① indicates the overall prevalence of dyslipidemia, ② indicates the prevalence of high total cholesterol (TC) levels, ③ indicates the prevalence of high triacylglycerol (TG) levels, ④ indicates the prevalence of high low-density lipoprotein (LDL) cholesterol levels, and ⑤ indicates the prevalence of low high-density lipoprotein (HDL) cholesterol levels.

**Table 2:** Quality assessment results of the included cross-sectional studies on the prevalence of dyslipidemia among Chinese teachers.

Included studies	Entry (in a dictionary, encyclopedia, etc.)									Score
	①	②	③	④	⑤	⑥	⑦	⑧	⑨	
Yao Xiezhen (15)	Y	Y	N	Y	Y	Y	Y	Y	Y	8
Ma Lihua (16)	Y	N	N	Y	Y	Y	Y	Y	Y	7
Dai Shuxian (17)	Y	N	N	N	Y	Y	Y	Y	Y	6
Jian Hu (18)	Y	N	N	Y	Y	Y	Y	N	Y	6
Jiao Zhiqin (19)	Y	Y	N	N	Y	Y	Y	Y	Y	7
Li Hong (20)	Y	N	N	Y	Y	N	Y	Y	Y	6
Zhang Guangbing (21)	Y	N	N	N	Y	Y	Y	Y	Y	6
Li Huajun (22)	Y	N	N	N	Y	Y	Y	Y	Y	6
Yun Wang (23)	Y	N	N	Y	Y	Y	Y	Y	Y	7
Jing Hong (24)	Y	N	N	Y	Y	N	Y	Y	Y	6
Cha Xiaojuan (25)	Y	N	N	N	Y	Y	Y	Y	Y	6
Ran Liang (26)	Y	Y	N	Y	Y	Y	Y	Y	Y	8

① Whether the sampling frame is suitable for the target population; ② Whether appropriate methods are adopted to select the participants; ③ Whether the sample size is sufficient; ④ Whether the participants and the study site are described in detail; ⑤ Whether there is sufficient coverage of the participants for data analysis; ⑥ Whether effective methods are adopted to identify the diseases or health problems; ⑦ Whether standard and credible methods are adopted to evaluate the subjects; ⑧ Whether the methods of data analysis are appropriate; ⑨ Whether the response rate is sufficient; if the response rate is low, the score is 0. Y represents 1 point for meeting the requirements. ⑩ Whether standard and credible methods were used to evaluate the study subjects; ⑪ Whether the method of data analysis was appropriate; ⑫ Whether the response rate was adequate, and if the response rate was low, whether appropriate methods were used to address the situation.

### 3.3 Meta-analysis results

#### 3.3.1 Analysis of the prevalence of dyslipidemia

**Figure 2:**
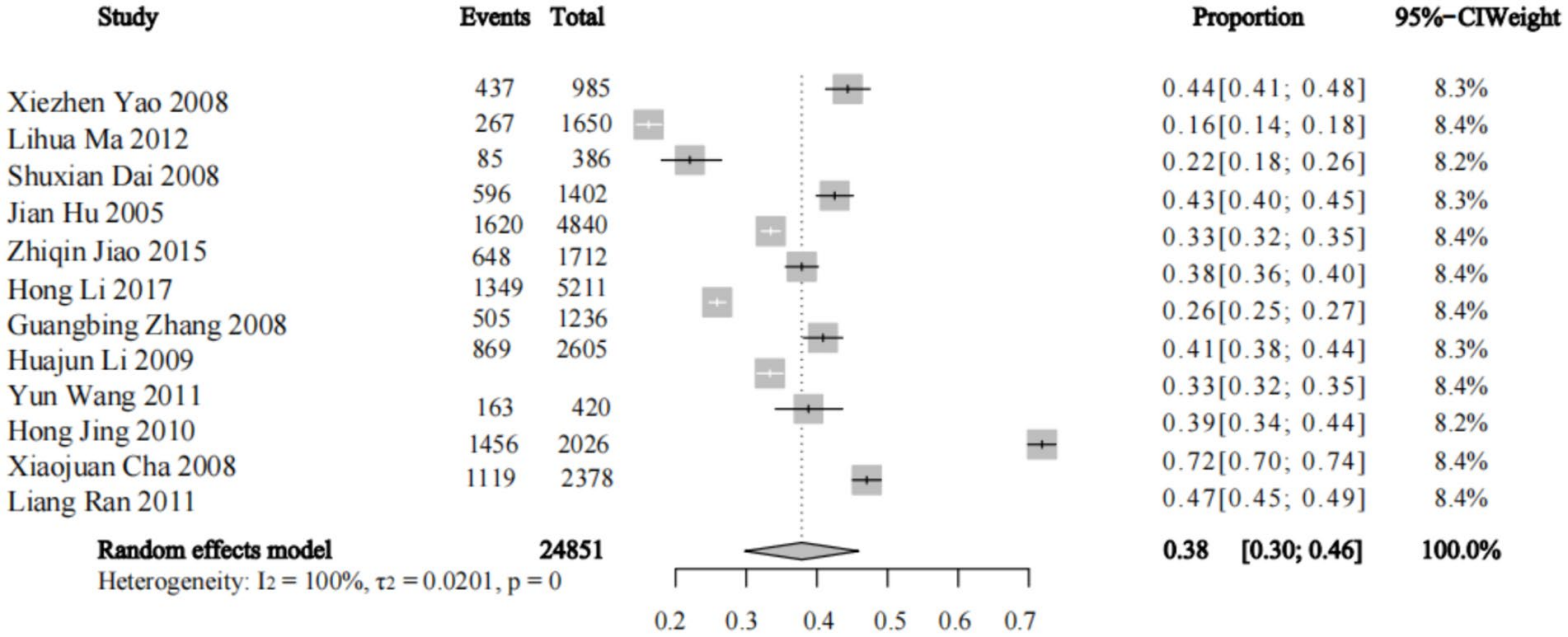
Forest plot of the meta-analysis data on the prevalence of dyslipidemia among teachers in China.

7 studies reported the prevalence of dyslipidemia, 5 studies reported the prevalence of hyperTG-emia, 3studies reported the prevalence of hyperTC-emia, 3 studies reported the prevalence of high LDL-C, and 4 studies reported the prevalence of low HDL-C. A meta-analysis using the random-effects model showed that the prevalence of dyslipidemia among teachers in China was 38% (*p* < 0.01) (Figure 2). In terms of the prevalence of different types of dyslipidemia, the prevalence of hyper-TGemia was 21.6% (*p* < 0.01), that of hyper-TCemia was 13.3% (*p* < 0.05), that of hyper-LDL-Cemia was 9.4% (p < 0.01), and that of hypo-HDL-Cemia was 4.3% (*p* = 0.25). There were differences in the prevalence of high TG, high TC and hyper-LDL-Cemia levels in terms of the prevalence of different types of dyslipidemia (*p* < 0.05) (Table 3).

**Table 3:** Meta-analysis results of the prevalence of different types of dyslipidemia among teachers in China.

Types of dyslipidemia	Number of included studies (items)	Heterogeneity of test results		general effects test		Prevalence (%)	95% CI
		I^2^ (%)	*p*	*Z*	*p*		
Dyslipidemia	7 (15–17, 20, 21, 24, 26)	92	*p* < 0.01	4.18	*p* < 0.0001	37	1.27∽1.95
Hypertriglyceridemia	5 (15, 16, 18, 22, 25)	76	*p* = 0.002	2.5	*p* = 0.01	21.5	1.05∽1.50
Hyper-TCemia	3 (16, 22, 25)	21	*p* = 0.28	1.75	*p* = 0.08	13.3	0.98∽1.34
Hyper LDL-Cemia	3 (15, 16, 22)	0	*p* = 0.90	2.30	*p* = 0.02	9.4	1.04∽1.59
Hypo-HDL-Cemia	4 (15, 18, 22, 25)	94	P < 0.01	1.15	P = 0.25	4.3	0.61∽6.52

#### 3.3.2 Subgroup analysis of the prevalence of dyslipidemia

**Figure 3:**
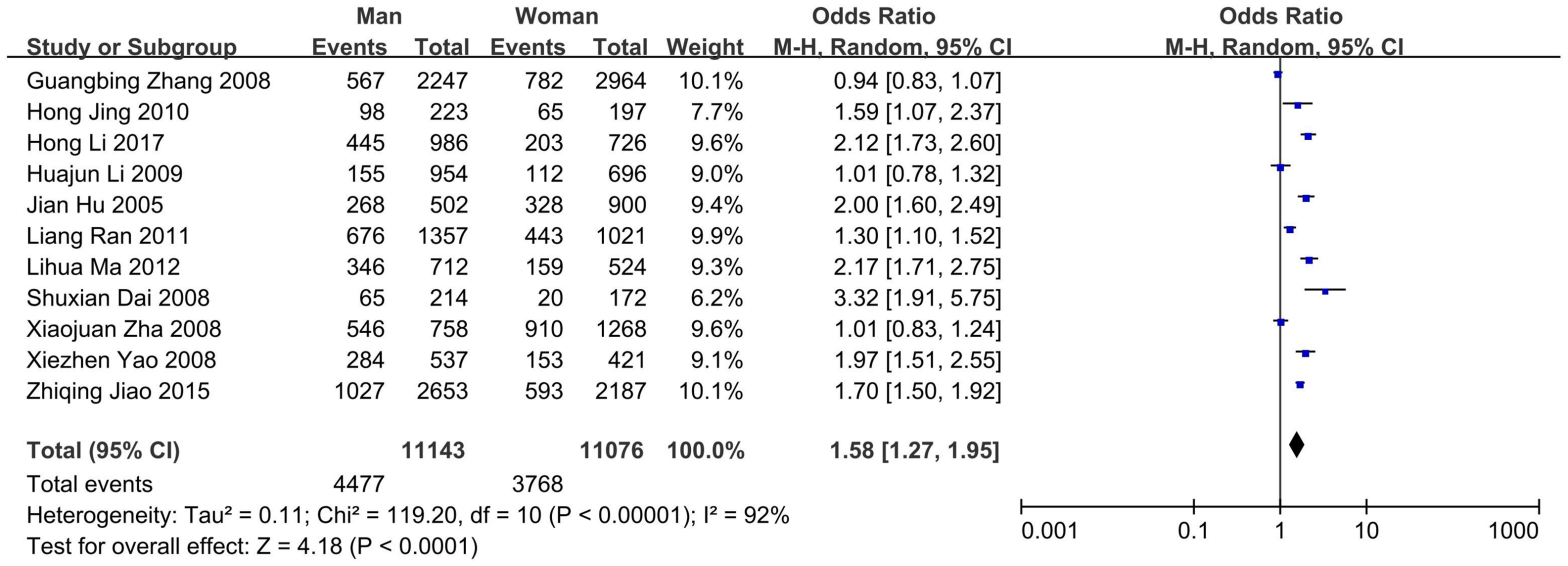
Meta-analysis results of the prevalence of dyslipidemia among teachers of the different sexes in China.

**Figure 4:**
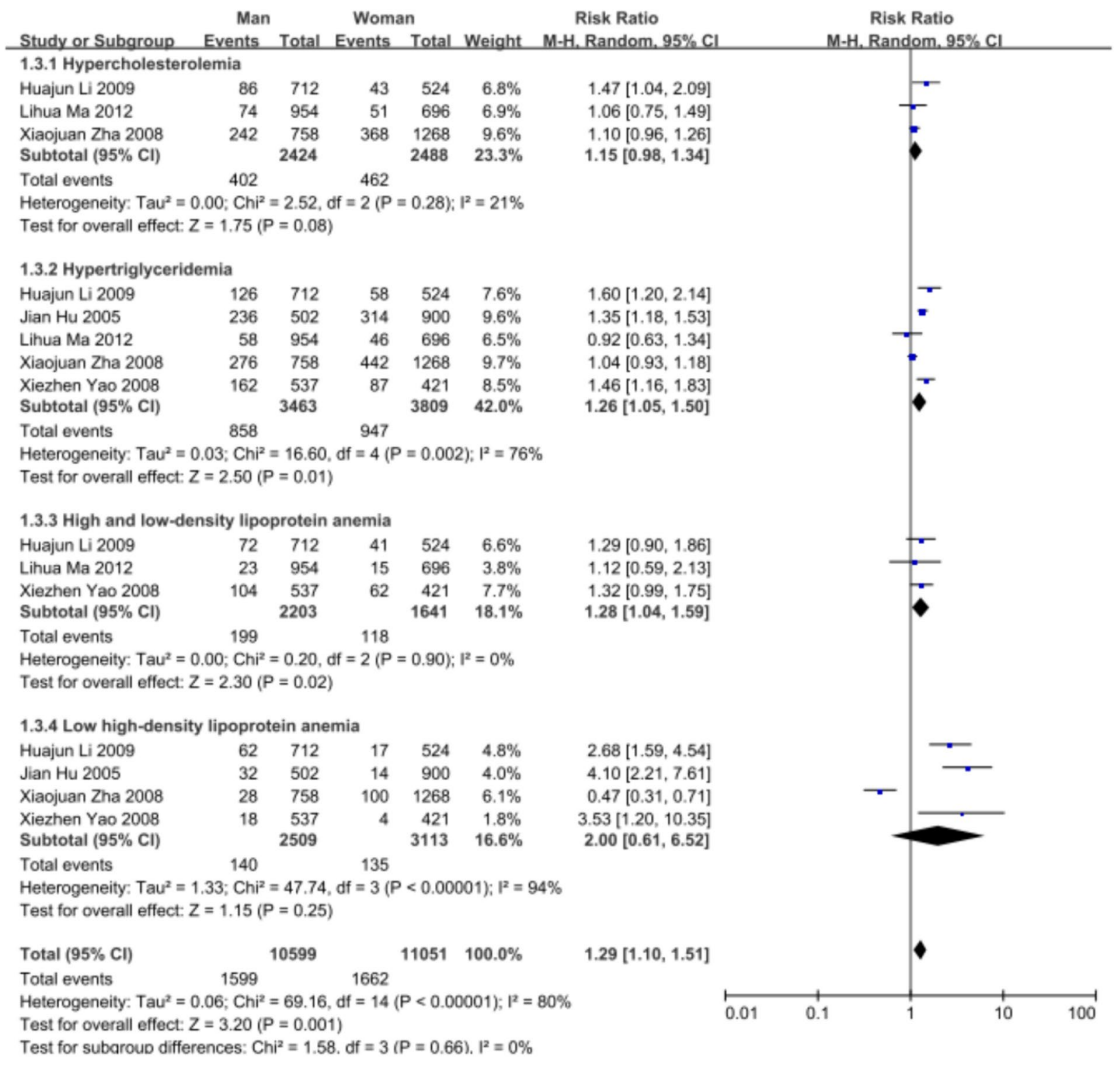
Meta-analysis results of the prevalence of different types of dyslipidemia among teachers in China.

**Figure 5:**
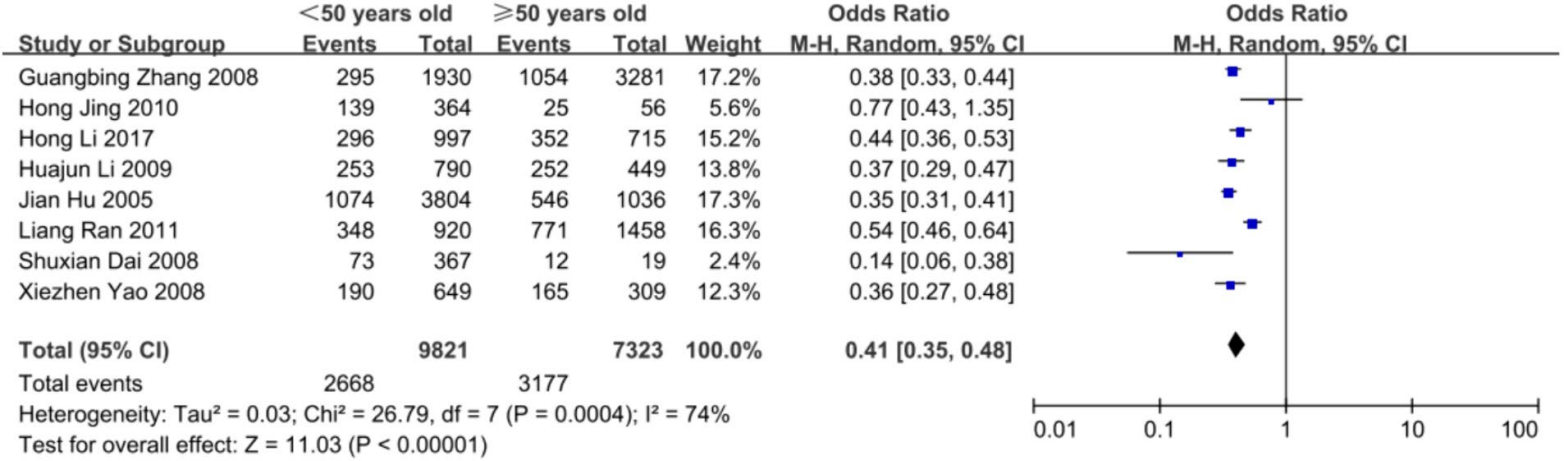
Meta-analysis results of the prevalence of dyslipidemia among teachers of different age groups in China.

Subgroup analysis was carried out using sex and age as subgrouping factors and a random-effects model. The results of the subgroup analysis were as follows. (1) In terms of sex, the heterogeneity of dyslipidemia among teachers of the different sexes was I^2^ = 92% (*p* < 0.01); female teachers differed from male teachers in terms of the overall prevalence of dyslipidemia and the prevalence of high TG and low H-LDC levels, with higher prevalence rates than those in male teachers, and there was no statistically significant difference in terms of the prevalence of high TC or high LDL-C levels (Figures 3, 4). (2) In terms of age, the heterogeneity of dyslipidemia among teachers of different ages was I^2^ = 74% (*p* < 0.01), and the risk of dyslipidemia was lower in teachers aged <50 years than in those aged ≥50 years (*p* = 0.004) (Figure 5).

#### 3.3.3 Influence factor regression analyses

To further search for sources of heterogeneity and to explore the factors influencing dyslipidemia, meta-regression analyses were performed for year of publication, region, and type of school. The results of the single influence factor analysis showed that different publication years (*p =* 0.77), different regions (*p* = 0.93), and different types of schools (*p = 0.18*) had no effects on the prevalence of dyslipidemia. Meta-regression analysis combining the above three factors showed consistent results with those of separate analyses, with no effect on the prevalence of dyslipidemia (I^2^ = 91.97%, *p* = 0.74), as shown in Table 4.

**Table 4:** Meta-regression analysis results of influencing factors.

Variable	Standard error	I^2^	P	Effect model	95% CI
Year of publication	0.02	94.00%	0.77	Random-effects model	−0.04∽0.05
Area	0.33	93.84%	0.93		−0.58∽0.73
School type	0.14	92.87%	0.18		−0.47∽0.90

#### 3.3.4 Publication bias

**Figure 6:**
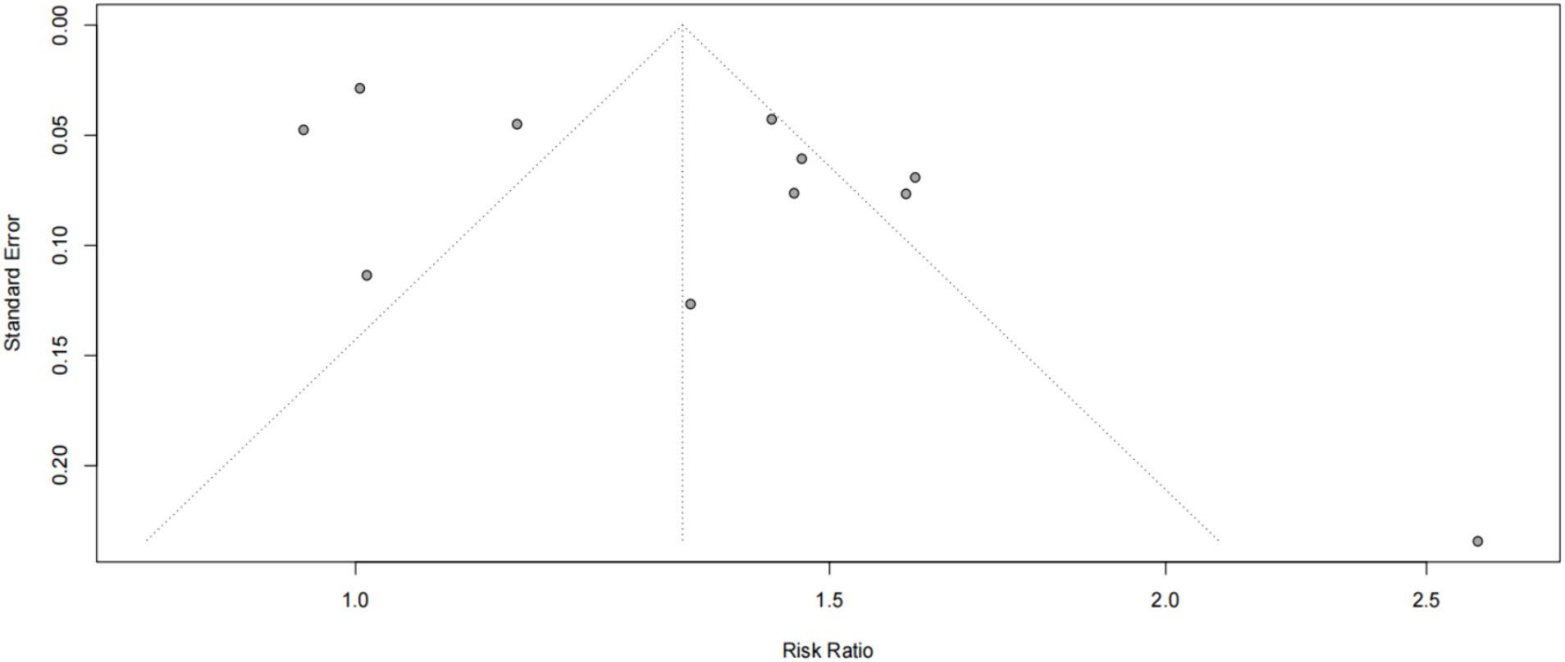
Publication bias plot for the included cross-sectional studies on the prevalence of dyslipidemia among teachers in China.

A funnel plot was used to analyze the publication bias for the included studies and showed that the study points were basically symmetrically distributed on both sides of the axis (see Figure 6), suggesting that the possibility of publication bias was small. Because one of the studies lacked data on dyslipidemia, only 11 articles are shown in the funnel plot for publication bias analysis. The Egger’s test results show (*t* = 1.37, *p* = 0.1702) that the possibility of publication bias is relatively small.

#### 3.3.5 Sensitivity analysis

**Figure 7:**
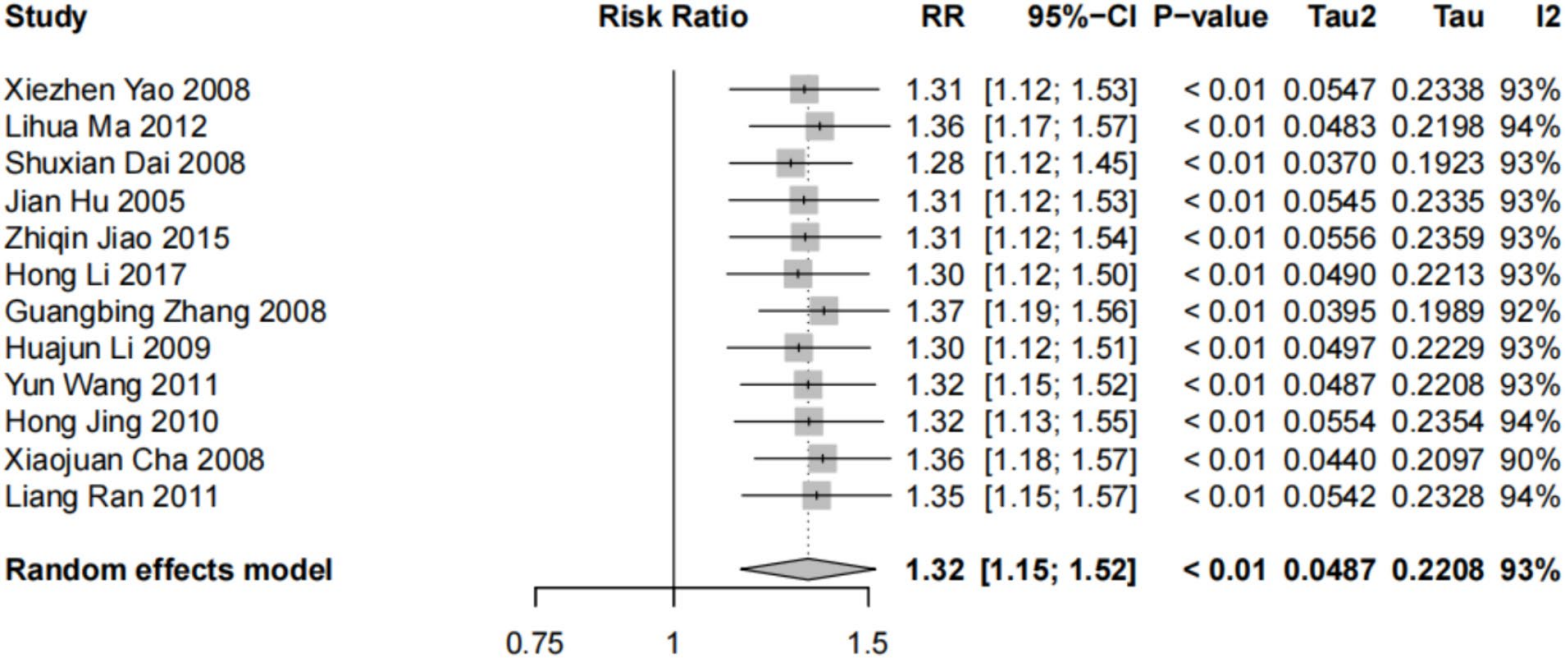
Sensitivity analysis of the included cross-sectional studies on the prevalence of dyslipidemia among teachers.

Sensitivity analyses were conducted by excluding individual studies one by one, and the results suggested that the stability of the results of the meta-analyses was good, as shown in Figure 7.

## 4 Discussion

### 4.1 The prevalence of dyslipidemia among teachers is greater than the national average

This study analyzed 12 cross-sectional studies from 11 provinces (municipalities directly under the central government) covering seven regions of China, namely, South China, East China, Central China, North China, Northeast China, Southwest China, and Northwest China, which included 24,851 teachers. The results of using the JBI article Evaluation Scale showed that the evaluation scores of the included studies were above 6 and that the quality of the article was of a medium-high grade. According to the results of the meta-analysis, the overall prevalence of dyslipidemia among teachers in China was 38%, which was higher than the national average of the prevalence of dyslipidemia among adults. Data from the 2018 national survey reported in the Report on Nutrition and Chronic Disease Situation of the Chinese Population (2020) (2) showed that the prevalence of dyslipidemia among adults aged 18 years or older was 35.6%. Dyslipidemia is a metabolism-related disease, the occurrence of which is strongly correlated with a poor lifestyle. High intake of sugar, fat and high-energy diets, alcohol consumption, obesity, staying up late and being sedentary are all risk factors. In the future, medical personnel should strengthen the health intervention and management of the teacher group, encourage them to actively participate in health testing and provide effective health education to improve their poor lifestyles. Educational management departments should pay attention to the health management and care of the teacher group, which can be done by appropriately adjusting the educational model settings, increasing the programs on physical activities for teachers, reducing the pressure of the profession and promoting the improvement of healthy lifestyles.

### 4.2 Prevalence of dyslipidemia among teachers in China and abroad

A study that evaluated the prevalence of metabolic syndrome among 1,165 Malaysian teachers revealed that overweight (obese) teachers accounted for 82.3% of the sample (27). A study of the health status of faculty members in some universities in the Arab world revealed a metabolic syndrome prevalence of more than 30% and a dyslipidemia and excess BMI prevalence with age (28). An assessment of changes in health risks among university staff undergoing long-term health interventions at Vanderbilt University in the United States revealed that the proportion of people with high cholesterol levels and high blood pressure had been on the rise since 2003 (29). A study by Sheffield Hallam University in the UK revealed no significant improvements in the BMI, systolic blood pressure, total cholesterol and HDL-C levels and body fat percentage over a five-year period in an overall analysis of five-year health data (30). It is clear that a range of metabolic disorders, such as dyslipidemia, blood pressure abnormalities and blood glucose abnormalities, are also prevalent in overseas teaching populations.

### 4.3 Sex subgroup analyses

To analyze the sources of heterogeneity in the prevalence of dyslipidemia among teachers, subgroup analyses and meta-regression analyses were performed for sex, age, region and school type. The overall prevalence of dyslipidemia and the prevalence of high TG and low HDL-C levels were greater in female teachers than in male teachers, and there were no statistically significant differences with regard to the prevalence of high TC and high LDL-C levels. The Report on Nutrition and Chronic Disease in China (2020) (2) shows that the overall prevalence of dyslipidemia in China is greater in men than in women and that the prevalence of dyslipidemia in men decreases with age, whereas in women, it increases with age. This may be related to the professional particularity of the teacher group, in which there is no difference in work content and work pressure between men and women; however, owing to the special characteristics of the female group, a decrease in estrogen levels after menopause can cause an increase in the activity of 3-hydroxy-3-methylglutaryl monoacyl-coenzyme a reductase (HMGR) in the liver, which in turn increases plasma cholesterol levels, thus triggering disorders of glucose and lipid metabolism in the organism (31). Therefore, our findings suggest that educational management organizations need to pay more attention to women’s physical and mental health in the workplace.

### 4.4 Age subgroup analysis

This study revealed that the prevalence of dyslipidemia among teachers aged <50 years was lower than that among teachers aged ≥50 years. Some studies in China have shown that a high detection rate of metabolism-related diseases is basically observed among teachers over 40 years old (32, 33), which is related to the physiological characteristics of the overall population as well as to the characteristics of the social class (34).

### 4.5 Meta-regression analyses for year, region, and school type

Analyses of the three dimensions, namely, the year of study publication, region, and school type stratum, revealed no significant differences in the prevalence of dyslipidemia. This may be related to factors such as the number of included studies and their design and the respondents and their access to information. For example, none of the studies reported whether there was a correlation between the level of education of teachers and the prevalence of dyslipidemia.

## 5 Conclusion

In conclusion, the prevalence of dyslipidemia in teachers in China is higher than the national average level, and there are differences in the prevalence of dyslipidemia in teachers of different genders and ages. Dyslipidemia is a metabolic related disease, and its occurrence has a great correlation with poor lifestyle, so it is necessary to strengthen the health management and intervention of teachers. Firstly, it is necessary to strengthen publicity and education, improve the health literacy of teachers, improve self-efficacy, and improve their health literacy and health compliance. Secondly, strengthen the construction of campus support system and improve the guarantee mechanism; Schools can be regarded as special functional communities to establish a group health management mechanism, improve the supervision and promotion role of partners through establishing partnerships, and effectively promote the change of health behaviors. Thirdly, improve the follow-up system, strengthen positive feedback, and strengthen positive behavior. Education management departments should attach importance to the health management of teachers. School can sign a contract with general practitioners, and closed-loop management of the group’s health schedule can be achieved through the establishment of follow-up and management of general practitioners.

## 6 Limitations

In conclusion, the prevalence of dyslipidemia among teachers is high in our country. There are differences in the prevalence of dyslipidemia among teachers from different sex and age groups. This study has certain limitations, as follows: (1) the included studies were cross-sectional studies, which are affected by the study design, sample selection, indicator measurement, and publication year, and publication bias is difficult to avoid; (2) some of the influencing factors, such as the age grading and education level, were not reported in the article included in the analysis, which may have had a certain impact on the results; and (3) the number of included studies was only 12, and the quality of some included studies was moderate. In the future, it is necessary to carry out a large-sample, multicenter epidemiological study to determine the prevalence of dyslipidemia in this group to validate the results of this study and, at the same time, provide a reference for good prevention and control of dyslipidemia-related chronic diseases.

## Data Availability

Data are available upon reasonable request to the corresponding author (dqf1689@smu.edu.cn), with Research Ethics Board approval.
