# Correction: Prevalence of dyslipidemia among teachers in China: a systematic review and meta-analysis

**DOI:** 10.3389/fpubh.2025.1666159

**Published:** 2025-07-30

**Authors:** Xiaoxue Wei, Feng Ouyang, Yang Liu, Qingfeng Du

**Affiliations:** ^1^Faculty of Medicine, Macau University of Science and Technology, Macau, China; ^2^The Seventh Affiliated Hospital of Southern Medical University, Foshan, China; ^3^School of Traditional Chinese Medicine, Southern Medical University, Guangzhou, China

**Keywords:** Chinese teachers, dyslipidemia, prevalence, meta-analysis, systematic review

Affiliation [^1^Faculty of Medicine, Macau University of Science and Technology, Macau, China] was erroneously given as [^1^School of Medicine, Macau University of Science and Technology, Macau, China].

The original version of this article has been updated.

