# Supplementary material for: Prevalence of dyslipidemia among teachers in China: a systematic review and meta-analysis
Source: Front Public Health. 2024 Aug 27;12:1425387. doi: 10.3389/fpubh.2024.1425387 (PMC11421384; doi:10.3389/fpubh.2024.1425387)
Supplement: Supplementary file 2 [file Table_2.DOC]

**Search Strategies**

**Pubmed:**

**#1： Search:** "School Teachers"[Mesh] Sort by: Most Recent

**#3： Search:** School Teacher[Title/Abstract] OR Teacher, School[Title/Abstract] OR Teachers, School[Title/Abstract] OR Middle School Teachers[Title/Abstract] OR Middle School Teacher[Title/Abstract] OR School[Title/Abstract] OR Teacher, Middle[Title/Abstract] OR School Teachers, Middle[Title/Abstract] OR Teacher, Middle School[Title/Abstract] OR Teachers, Middle School[Title/Abstract] OR Elementary School Teachers[Title/Abstract] OR Elementary School Teacher[Title/Abstract] OR School Teacher, Elementary[Title/Abstract] OR School Teachers, Elementary[Title/Abstract] OR Teacher, Elementary School[Title/Abstract] OR Teachers, Elementary School[Title/Abstract] OR High School Teachers[Title/Abstract] OR High School Teacher[Title/Abstract] OR School Teacher, High[Title/Abstract] OR School Teachers, High[Title/Abstract] OR Teacher, High School[Title/Abstract] OR Teachers, High School[Title/Abstract] OR Pre-School Teachers[Title/Abstract] OR Pre-School Teacher[Title/Abstract] OR Pre School Teachers[Title/Abstract] OR Teacher, Pre-School[Title/Abstract] OR Teachers, Pre-School[Title/Abstract]

**#4： Search:** "Dyslipidemias"[Mesh] Sort by: Most Recent

**#5： Search:** Dyslipidemia[Title/Abstract] OR Dyslipoproteinemias[Title/Abstract] OR Dyslipoproteinemia[Title/Abstract]

**#6： Search:** ((("School Teachers"[Mesh]) OR (School Teacher[Title/Abstract] OR Teacher, School[Title/Abstract] OR Teachers, School[Title/Abstract] OR Middle School Teachers[Title/Abstract] OR Middle School Teacher[Title/Abstract] OR School[Title/Abstract] OR Teacher, Middle[Title/Abstract] OR School Teachers, Middle[Title/Abstract] OR Teacher, Middle School[Title/Abstract] OR Teachers, Middle School[Title/Abstract] OR Elementary School Teachers[Title/Abstract] OR Elementary School Teacher[Title/Abstract] OR School Teacher, Elementary[Title/Abstract] OR School Teachers, Elementary[Title/Abstract] OR Teacher, Elementary School[Title/Abstract] OR Teachers, Elementary School[Title/Abstract] OR High School Teachers[Title/Abstract] OR High School Teacher[Title/Abstract] OR School Teacher, High[Title/Abstract] OR School Teachers, High[Title/Abstract] OR Teacher, High School[Title/Abstract] OR Teachers, High School[Title/Abstract] OR Pre-School Teachers[Title/Abstract] OR Pre-School Teacher[Title/Abstract] OR Pre School Teachers[Title/Abstract] OR Teacher, Pre-School[Title/Abstract] OR Teachers, Pre-School[Title/Abstract])) AND ("Dyslipidemias"[Mesh])) AND (Dyslipidemia[Title/Abstract] OR Dyslipoproteinemias[Title/Abstract] OR Dyslipoproteinemia[Title/Abstract])

**Web of Science:**

title "teacher*" (match all words) and abstract or title "Dyslipidemias" (match all words)

**CINAHL Ultimate：**

SU 教师 OR SU 教职工 OR SU 老师 AND 血脂

**Chinese database：**

#1: 老师

#2: 教师

#3: 教职工

#4: 血脂

#5: 高血脂

#6: 血脂异常

#7: 健康状况

#8:中国

#9: #1OR#2OR#3AND#4OR#5OR#6OR#7AND#8
